# Using the experience‐based co‐design method to design a teaching package

**DOI:** 10.1111/medu.13720

**Published:** 2018-10-21

**Authors:** Julie L Darbyshire, Lisa Hinton, J Duncan Young, Paul R Greig

## What problems were addressed?

The World Health Organization recommends average noise levels in patient care areas no higher than 35 dB, with peaks below 40 dB. In reality, most intensive care units (ICUs) worldwide average 55‐60 dB, with peaks up to 120 dB. Staff acclimatise to this, often rationalising high noise levels as necessary because of the equipment and monitoring needs of patients. There is limited awareness that many sources of noise are modifiable, understanding of the patient experience of intensive care is low, and prior to training there is little motivation to implement change. The objective of this training was to facilitate reflection on the patient experience, with a view to enabling self‐directed change to reduce noise levels in the ICU.

## What was tried?

Building on interviews with former ICU patients^1^ and ethnographic observations, we used the experience‐based co‐design method to create a training course to increase knowledge about noise in the ICU. A collaboration between patients, clinical staff, researchers and medical educationalists ensured learning objectives were relevant, practical and of high educational quality.

The course combines a multimedia e‐learning package and an *in situ* simulated patient experience that combines a binaural soundtrack of ICU noises with live‐action nursing activities. The e‐learning takes approximately 20 minutes to complete and individual experiences last 15 minutes, which includes post‐experience debriefing. The authenticity of teaching materials is ensured by using real patient experiences and replicating behaviours observed during ethnography sessions.

An online self‐assessment is included because formative assessment is both motivational and guides onward learning. Assessment points were mapped to course materials using a curriculum blueprint, and participant feedback was used to refine the simulation experience.

## What lessons were learned?

The simulation invoked feelings of worry, fear, stress, confusion and loneliness. This maps well to patient narratives and indicated our simulation had been successful despite only lasting a few minutes. Post‐experience debriefing allowed participants to decompress before returning to work. The negative emotional component was intended to prompt staff reflection, as per Kolb's learning cycle, and proved effective. All 116 participants described the experience as useful, >90% reporting they should do more to improve the environment for their patients.

After the experience, 97% of staff indicated they would modify their behaviours, and this appeared to be carried into practice. Noise level monitoring confirmed a perceivable drop of ~4 dB from a baseline of 57.0 to 53.2 dB during the 4‐month post‐intervention period.

We have been unable to incorporate this training into recurring education sessions. The *in situ* experience is heavily workload dependent, requiring a bed space and members of the education team to be free at an appropriate time. This requires a flexible, imaginative and highly motivated faculty member to deliver training opportunistically; it was therefore difficult to embed. We are now exploring alternative models to enable stand‐alone delivery of this innovative training.

We have been able to demonstrate that meaningful teaching can be delivered even in a tightly compressed time format, and that this can successfully modify behaviour. This makes this style of teaching practical in a busy unit, but maintaining momentum after the initial wave of interest remains challenging.
